# Amphibian diversity in Serranía de Majé, an isolated mountain range in eastern Panamá

**DOI:** 10.3897/zookeys.859.32869

**Published:** 2019-07-02

**Authors:** Daniel Medina, Roberto Ibáñez, Karen R. Lips, Andrew J. Crawford

**Affiliations:** 1 Laboratório de História Natural de Anfíbios Brasileiros (LaHNAB), Departamento de Biologia Animal, Universidade Estadual de Campinas, Campinas, SP 13083-862, Brazil Smithsonian Tropical Research Institute Panamá Panama; 2 Smithsonian Tropical Research Institute, Apartado Postal 0843-03092, Panamá, República de Panamá Universidade Estadual de Campinas Campinas Brazil; 3 Círculo Herpetológico de Panamá, Estafeta Universitaria, Apartado Postal 10762, Panamá, República de Panamá Estafeta Universitaria Panamá Panama; 4 Departamento de Zoología, Universidad de Panamá, Panamá, República de Panamá Universidad de Panamá Panamá Panama; 5 Sistema Nacional de Investigación, Panamá, República de Panamá Sistema Nacional de Investigación Panamá Panama; 6 Department of Biology, University of Maryland, College Park, MD 20742, USA University of Maryland College Park United States of America; 7 Departamento de Ciencias Biológicas, Universidad de los Andes, Bogotá, 111711, Colombia Universidad de los Andes Bogotá Colombia

**Keywords:** Altitudinal diversity, amphibian species inventory, Panamá

## Abstract

Eastern Panamá is within the Mesoamerican biodiversity hotspot and supports an understudied amphibian fauna. Here we characterize the amphibian diversity across an elevational gradient in one of the least studied mountain ranges in eastern Panamá, Serranía de Majé. A total of 38 species were found, which represent 17% of all species reported for Panamá. Based on expected richness function and individual-based rarefaction curves, it is estimated that this is an underestimate and that at least 44 amphibian species occur in this area. Members of all three amphibian orders were encountered, represented by ten families and 22 genera, including five species endemic to Central America. Estimated species richness decreased with elevation, and the mid-elevation site supported both lowland and highland species. Our study provides a baseline for understanding the distribution pattern of amphibians in Panamá, for conservation efforts, and for determining disease-induced changes in amphibian communities.

## Introduction

Mesoamerica is a global biodiversity hotspot (Johnson et al. 2015). Within this region, Panamá has the second greatest number of reptile and amphibian species, containing 26% of all amphibian species reported for Mesoamerica (Jaramillo et al. 2010). However, a substantial portion of eastern Panamá has been understudied. Geographically, eastern Panamá comprises the northernmost part of the Chocó biogeographical region (Duque-Caro 1990), and it is part of the Tumbes-Chocó-Magdalena global biodiversity hotspot (Mittermeier et al. 1999). This region includes a number of relatively low mountain ranges, including the Serranía de San Blas + Serranía del Darién on Caribbean side, the inland Serranía de Pirre + Altura de Nique + Altos de Quía, Serranía de Majé, and the Serranía de Sapo + Serranía de Jingurudó + Altos de Aspavé + Cordillera de Juradó along the Pacific Ocean (Duque-Caro 1990; Batista et al. 2016).

Whitfield et al. (2016) analyzed regional trends and reported that eastern Panamá has a very small number of recognized species in relation to its geographic area, which reflects the limited number of field surveys in the area. A sharp increase in the number of field surveys during the last decade has led to the discovery of several new amphibian species with restricted distribution ranges (e.g., Ibáñez and Crawford 2004; Crawford et al. 20104a; Batista et al. 2014a, 2014b, 2016) supporting the hypothesis that eastern Panamá is a region with a high endemic amphibian diversity. This is in contrast to the claim that it was mainly a dispersal route during the Great American Biotic Interchange (Webb 2006), and was colonized by species groups from the north and South America (Vanzolini and Heyer 1985; Pinto-Sánchez et al. 2012).

One reason to establish baseline estimates of amphibians is to assess changes following loss caused by disease epidemics. The pathogenic fungus *Batrachochytriumdendrobatidis* (*Bd*) causes population declines and extinctions of many amphibian species worldwide, particularly in the Neotropics (James et al. 2015, Lips 2016). *Bd* has caused dramatic declines of amphibian communities in the highlands of western and central Panamá (Lips 1999; Lips et al. 2006; Crawford et al. 2010b). Importantly, to our knowledge, at the time of sampling there were no published data reporting the presence of *Bd* in the region – though the amphibian species present at this region can either represent the original community or a subset as a consequence of an undetected *Bd* epidemic. Here, we describe the results from field surveys to characterize α and β diversity along an altitudinal gradient in the isolated Serranía de Majé of eastern Panamá.

## Materials and methods

### Study sites

**Figure 1.**
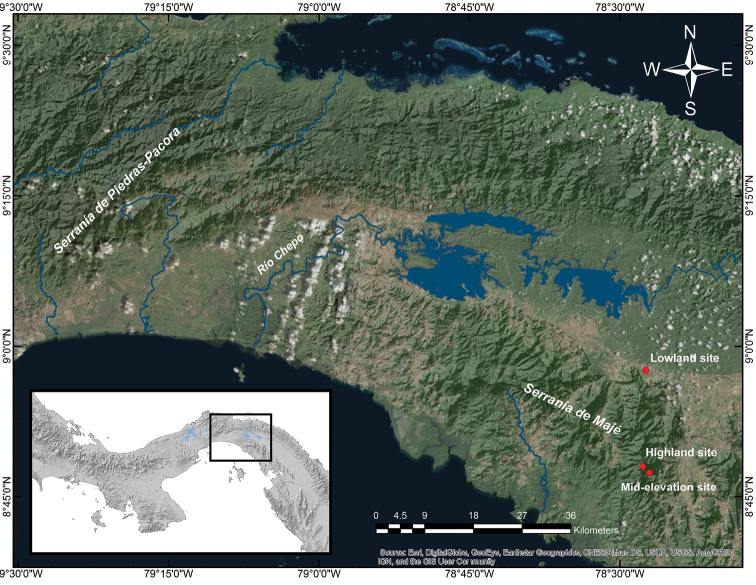
Map showing the location of the study sites in the Serranía de Majé and the Serranía de Piedras-Pacora across the valley of the Chepo River.

During the wet season, from June 23 to July 2 2007, we conducted field surveys at three study sites located at a low, middle, and high elevations in the Serranía de Majé. This mountain range is located on the Pacific coast, previously known as Serranía de Cañazas (Myers 1969), and is isolated from others mountainous areas by the Chepo and Chucunaque Rivers (Figure 1; Angehr and Christian 2000). Its highest point, Cerro Chucantí (1,489 m), stands on the eastern end of the mountain range, at the boundary of the Panamá and Darién provinces (Angher and Christian 2000).

The three study sites were located in Lowland Wet/Moist Forest (LWM) below 600 m, and Premontane Rain Forest/Wet Forest (PRW) above 600 m (Holdridge 1967). The sites were: a low elevation site, Centro Cristo Misionero (8.96N, 78.457W) at 120–150 m elevation; a mid-elevation site, located within the Reserva Natural Privada Cerro Chucantí (8.79N, 78.451W) at 797 m elevation; and, the high elevation site, also located in the Cerro Chucantí private natural reserve (8.80N, 78.462W), near the top of the Cerro Chucantí, at 1,240–1,365 m elevation. The approximate airline distances between the study sites were 19, 18, and 2 km for lowland-mid-elevation, lowland-highland and mid-elevation-highland sites, respectively.

### Data collection

The surveys were conducted using the sampling technique “free and unrestricted search”, which is considered to be one of the most efficient methods to record a high number of species in a relatively short amount of time (Rueda et al. 2006). Different types of habitat such as forest, streams, ponds, and open areas with grass were surveyed during the day and night. Species identification and individual counts were performed using the techniques ‘visual encounter survey’ (VES) and ‘acoustic encounter survey’ (AES). In addition, the search effort invested (in person-hours) at each sampling site was calculated by multiplying the search time by the number of observers, and the catch per unit of search effort for each site was calculated by dividing the number of post-metamorphic amphibians encountered by the search effort at the respective site as estimated by Kilburn et al. (2011).

A few specimens of each species were collected as voucher specimens (Suppl. material 1: Table S1), photographed, and deposited in the reference Collection of Herpetology (specimen tags CH and AJC) at the Smithsonian Tropical Research Institute, and in the Museo de Vertebrados de la Universidad de Panamá (tags MVUP). Amphibians to be preserved were first euthanized using Orajel (benzocaine 20%) or occasionally 10% ethanol. Before fixation, liver samples were taken from each specimen and preserved for future phylogenetic and phylogeographic analyses (Seutin et al. 1991). Vouchers were then fixed in 10% formalin in a position that facilitates examination. To verify the identification of specimens we used all relevant literature available on the amphibians of Panamá (e.g., Ibáñez et al. 1999a), and compared specimens with those in the CH reference collection. The identification of anuran advertisement calls was facilitated by audio recordings of Panamanian frogs (Ibáñez et al. 1999b).

### Data analyses

We calculated α diversity based on all post-metamorphic amphibians captured at each site (Hortal et al. 2006), using the software EstimateS 8.0.0 (Colwell 2006). We calculated Mao Tau (Colwell et al. 2004) and plotted sample-based rarefaction curves with 95% confidence intervals.

To determine β diversity for assessing the variation in species composition across sites, we also used all post-metamorphic amphibians captured at each site, and conducted a cluster analysis based on Jaccard dissimilarity measures estimated with the R function vegdist from the *vegan* package (Oksanen et al. 2017). In order to identify clusters, we built a dendrogram using the unweighted pair-group method based on arithmetic averages (UPGMA), using function hclust from the default R package *stats*. This analysis was completed in the R version 3.3.3 (R Core Team, 2017).

## Results

Our team conducted 280 person-hours of surveys (lowland site: 125; mid-elevation site: 96; highland site: 59) and identified 38 amphibian species from all three amphibian orders, ten families, and 22 genera (Table 1). The total number of species for the surveyed area within the Serranía de Majé was estimated as 44 species based on the upper 95% confidence interval of the Mao Tau function (Table 2).

**Table 1.** List of species and number of post-metamorphic individuals found at the three surveyed sites across the elevational gradient in the Serranía de Majé. The letter ‘L’ refers to a species that was recorded by its larvae and ‘V’ by its vocalizations. The IUCN conservation status is based on the IUCN (2018). ‘E’ represents a species that is endemic to Central America (CA) based on Johnson et al. (2015).

Order	Family	Genus	Species	Lowland	Mid-elevation	Highland	IUCN status	Endemic to CA
** Anura **	Aromobatidae	* Allobates *	* talamancae *	2	1		LC	
	Bufonidae	* Rhaebo *	* haematiticus *		9	11	LC	
		* Rhinella *	* alata *	13	1	1	LC	
		* Rhinella *	* horribilis *	2	1		LC	
	Centrolenidae	* Espadarana *	* prosoblepon *	L	8	V	LC	
		* Cochranella *	* euknemos *		3		LC	
		* Hyalinobatrachium *	* colymbiphyllum *	1			LC	
		* Hyalinobatrachium *	* fleischmanni *	3			LC	
		* Hyalinobatrachium *	* vireovittatum *		1		DD	E
	Craugastoridae	* Craugastor *	* crassidigitus *	1	9	1	LC	
		* Craugastor *	* fitzingeri *	5	3		LC	
		* Craugastor *	* raniformis *	15	3		LC	
		* Pristimantis *	aff. latidiscus			4	–	–
		* Pristimantis *	* caryophyllaceus *			57	NT	E
		* Pristimantis *	* cruentus *		1	71	LC	
		* Pristimantis *	* gaigei *	1			LC	
		* Pristimantis *	* moro *			10	LC	
		* Pristimantis *	* pardalis *			1	NT	E
		* Pristimantis *	* ridens *		1		LC	
		* Pristimantis *	* taeniatus *		V		LC	
		* Strabomantis *	* bufoniformis *	2			LC	
	Dendrobatidae	* Colostethus *	aff. pratti	11	9	4	–	–
		* Dendrobates *	* auratus *	8	19		LC	
		* Silverstoneia *	aff. nubicola	3	12	4	–	–
	Eleutherodactylidae	* Diasporus *	aff.diastema*	21			–	–
		* Diasporus *	majeensis**			1	–	E
	Hylidae	* Agalychnis *	* callidryas *	L	4		LC	
		* Dendropsophus *	* microcephalus *	10			LC	
		* Boana *	* rosenbergi *	4			LC	
		* Scinax *	* rostratus *	2			LC	
		* Scinax *	* ruber *	3			LC	
		* Smilisca *	* phaeota *	6			LC	
		* Smilisca *	* sila *		12		LC	
	Leptodactylidae	* Engystomops *	* pustulosus *	13	1		LC	
		* Leptodactylus *	* fragilis *	3			LC	
		* Leptodactylus *	* savagei *	1	V		LC	
** Caudata **	Plethodontidae	* Oedipina *	* complex *			1	LC	
** Gymnophiona **	Caeciliidae	* Caecilia *	* isthmica *		1		DD	E
**3**	**10**	**22**	**38**	**130**	**99**	**166**		

^*^ This species refers to the Diasporusaff.diastema from the Serranía de Majé as suggested by Batista et al. (2016). ^**^ Described by Batista et al. (2016), only known from Panamá; therefore, considered endemic to CA.

The greatest number of species was found at the lowland site (24 spp., Table 2; individuals catch per unit of search effort: 1.04), where the search effort was the highest, and where multiple aquatic habitats were available (i.e. ponds and forest streams). The most abundant species at this site were Diasporusaff.diastema (sensu Batista et al. 2016), *Craugastorraniformis* and *Engystomopspustulosus* (Table 1). *Espadaranaprosoblepon* and *Agalychniscallidryas* were detected at this site with larval surveys. The mid-elevation site had fewer species than the lowland site (22 spp., Table 2; individuals catch per unit of search effort: 1.03); however, despite lower search effort at this site, the upper 95% confidence intervals of the Mao Tau function estimated very similar species number (i.e., 25 spp. at the lowland site and 26 spp. at the mid-elevation site). The most abundant species at the mid-elevation site were *Dendrobatesauratus*, Silverstoneiaaff.nubicola and *Smiliscasila* (Table 1). In addition, at this site two species were detected only by their vocalizations: *Pristimantistaeniatus* and *Leptodactylussavagei*. The lowest richness was observed at the highland site (13 spp., Table 2; frog catch per unit of search effort: 2.81), because of limited searching effort, fewer habitats, and the lower diversity of the upland area. The estimated number of species for this site based on the upper 95% confidence intervals of the Mao Tau function was 17 spp., and the most abundant species at this site were *Rhaebohaematiticus*, *Pristimantiscaryophyllaceus*, *P.cruentus*, and *P.moro* (Table 1). Moreover, the glassfrog, *Espadaranaprosoblepon*, was detected at this site only by its vocalization.

**Table 2.** Total number of post-metamorphic individuals and species per site, and the site-level estimated richness as a function of the 95% confidence intervals (CI) calculated by the function Mao Tao.

Site	Number of Individuals	Number of species observed (Sobs)	Expected 95% CI upper limit
**Lowland**	130	22	24.64
**Mid-elevation**	99	19	25.58
**Highland**	166	12	16.57
**All sites**	395	37	44.08

**Figure 2.**
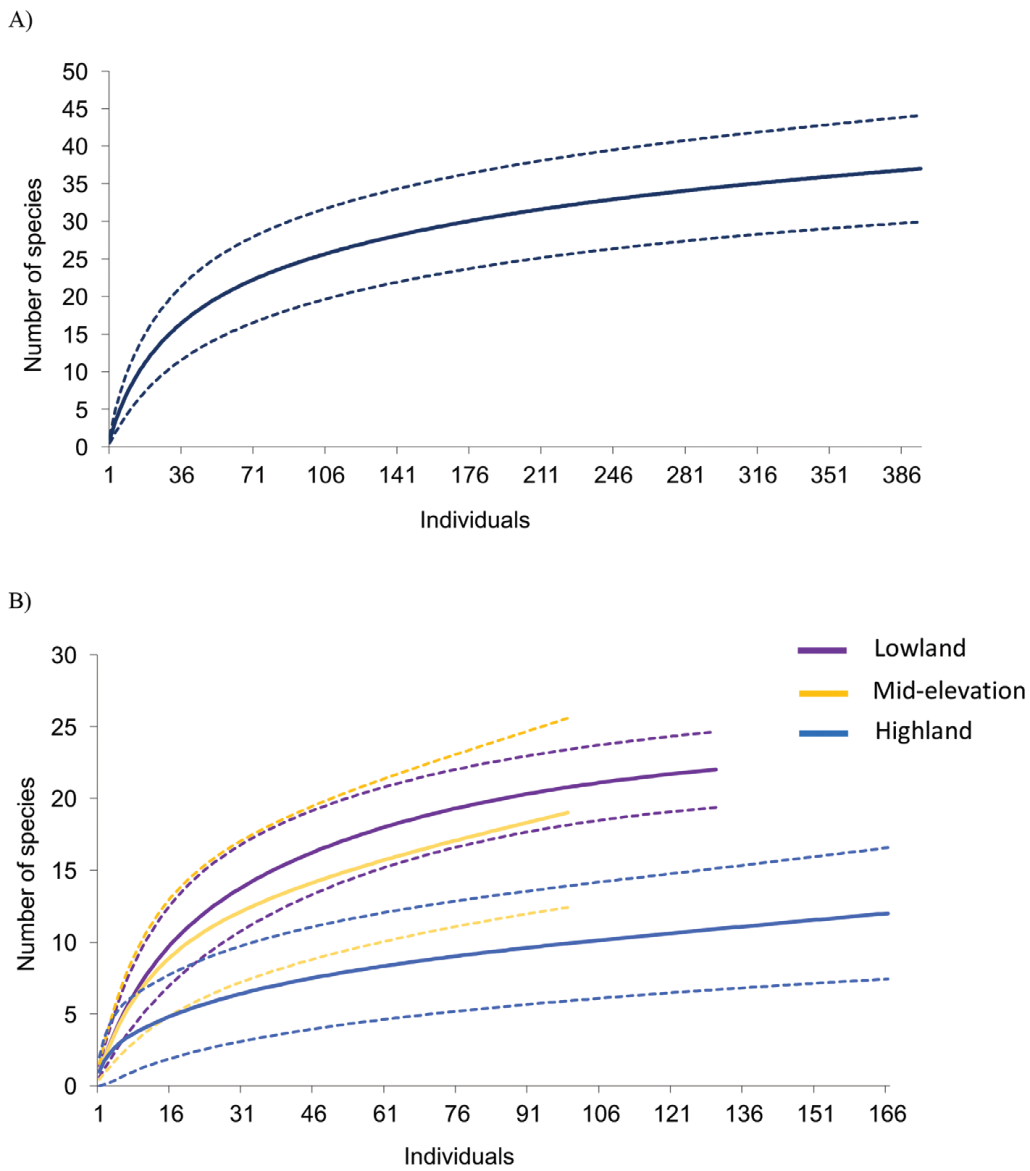
Individual-based rarefaction curves showing the estimated richness as a function of the upper 95% confidence interval (CI) calculated by the function Mao Tao. **A** Rarefaction curve combining all data obtained for the Serranía de Majé transect **B** rarefaction curves for low (120 – 150 m), intermediate (797 m), and high elevation (1,240–1,365 m) survey sites.

The individual-based rarefaction curves for the total area surveyed (Figure 2A) and at the site level (Figure 2B) showed a substantial decrease in the slope as the number of individuals increased with search effort. Thus, while the upper 95% confidence interval of the Mao Tau function suggests that not all species present in the area were observed, the amphibian community determined in these surveys might be representative of the extant community in Serranía de Majé.

**Figure 3.**
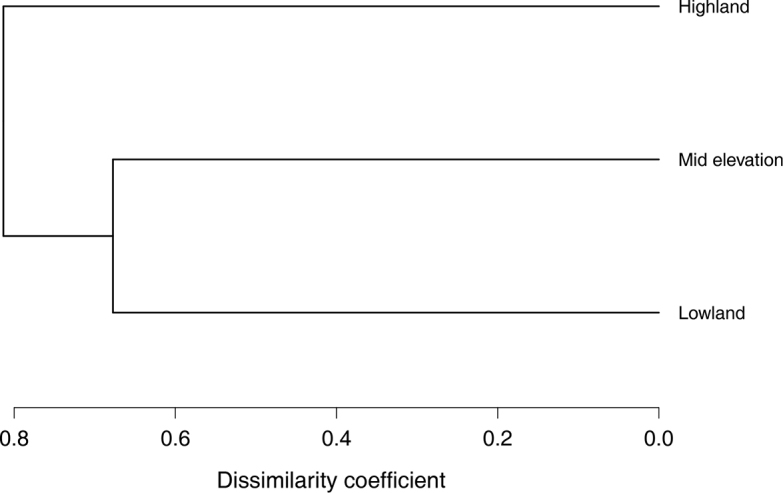
Site-level dendrogram based on Jaccard dissimilarities and built with the unweighted pair-group method based on arithmetic averages (UPGMA). This analysis was based on all post-metamorphic amphibians captured at each site.

Based on the Jaccard dissimilarity coefficients calculated, the community composition was more similar between the low and mid-elevation sites relative to the high elevation site (Table 3, Figure 3). As expected, the sites that most differed were the low versus high elevation sites. However, six species were consistently present across all three elevation sites: *Rhinellaalata*, *Espadaranaprosoblepon*, *Craugastorcrassidigitus*, Colostethusaff.pratti, and Silverstoneiaaff.nubicola.

**Table 3.** Number of species shared between pairs of sites along an elevational transect of the Serranía de Majé (below the diagonal); total number of species per site including the species registered by post-metamorphic stages, vocalization, or larval stage (**diagonal**); and Jaccard similarity coefficients (1 - dissimilarity estimate) for each pair of sites (*above the diagonal*).

	Lowland	Mid-elevation	Highland
**Lowland**	**24**	*0.32*	*0.13*
**Mid-elevation**	13	**22**	*0.24*
**Highland**	5	7	**13**

## Discussion

The present study represents the first attempt to characterize the composition and altitudinal diversity pattern of the amphibian community from the isolated Serranía de Majé of eastern Panamá. We determined that the composition of the species community across the altitudinal gradient was comprised by species from both Mesoamerican and South American groups, and that taxonomic genera from South America dominated the composition of the community (South American genera: 82%; Mesoamerican genera: 18%). In addition, the observed proportion in the composition of genera is consistent with the diversity pattern determined by Savage (2002) for eastern Panamá, where genera from South American groups represented over 50% of the genera comprising the amphibian assemblage.

The species found during this study represent 17% of the native amphibian species of Panamá (AmphibiaWeb 2018). However, the estimated total number of species based on the rarefaction analysis suggests that the richness of the study area is slightly higher than what we observed. In addition, the recent discovery of two new amphibian species from the Serranía de Majé, *Bolitoglossachucantiensis* and *Diasporusmajeensis* (see Batista et al. 2014b, 2016), suggest that this region might be high in endemism, as previously suggested for eastern Panamá (Crawford et al. 2010a).

Amphibians, occurring in Central America, have their highest species richness at intermediate elevations (Savage 2002, Wiens et al. 2006, Whitfield et al. 2016). This general altitudinal diversity pattern might also apply to the Serranía de Majé considering that, despite the relatively lower search effort and lower number of individuals encountered at the mid-elevation site, we observed and estimated a species richness similar to that of the lowland site (i.e., site with the highest observed richness). In addition, we determined similar estimates of individuals catch per unit of search effort (lowland site: 1.04 vs. mid-elevation site:1.03) between the lowland and mid-elevation sites. Hence, these results suggest that an increase in sampling effort at the mid-elevation site will potentially increase the number of species detected. Lastly, the observed altitudinal pattern of species richness could have been influenced by the variation across sites in the area covered during the surveys and the habitat types present at the sampling sites. In particular, the number of observed species at the highland site was potentially affected by the absence of streams and ponds, and the reduced patch size of the cloud forest.

In terms of β diversity, the higher similarity in the community composition between the mid-elevation and highland sites compared to that between the lowland and highland sites, suggests that the composition at intermediate elevations in Serranía de Majé might result, in part, by an overlap in the altitudinal distribution of the species associated with higher and lower altitudes; a pattern previously observed for the anuran communities from the Panamá Canal watershed (Ibáñez et al. 2002). In addition, despite the mid-elevation and highland sites being closer to each other (i.e., ~2 km apart) than to the lowland site, the community composition between the mid-elevation and highland sites was less similar than the composition between the mid-elevation and lowland sites. The higher similarity in the community composition between the mid-elevation and lowland sites compared to that with the highland site suggests that the highland site might be comprised by species with restricted altitudinal distributions. For instance, the dissimilarity associated with the highland site in our study was potentially influenced by the observation of species with restricted altitudinal ranges, such as: Pristimantisaff.latidiscus, *P.caryophyllaceus*, *P.moro*, *P.pardalis* and *Diasporusmajeensis*.

**Figure 4.**
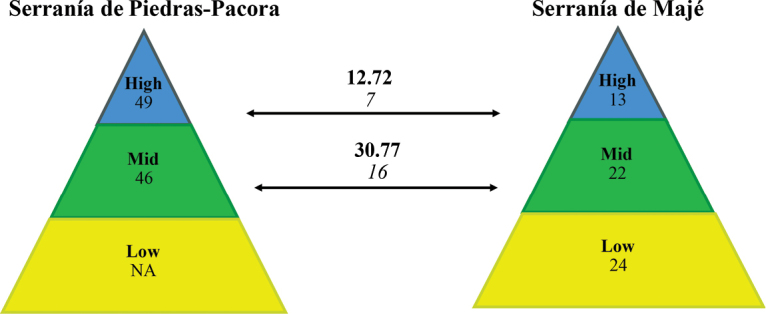
Diagram showing a decrease with elevation in the similarities of amphibian species assemblages associated with sites from the Serranía de Piedras-Pacora mountain range and the isolated Serranía de Majé mountain range. The numbers represent the shared species between sites (*N*), Jaccard similarity coefficients (**N**) and total number of species at the site level (N). Each color represents an elevation category, where the lowlands (< 400 m) are represented in yellow, mid-elevation sites (400–800 m) in green, and highlands (> 800 m) in blue. NA = no data available.

The Serranía de Majé is isolated from the other mountain ranges in the region by the valleys of the Chepo and Chucunaque Rivers (Figure 1), which could have represented physical barriers leading to genetic isolation of populations that could have resulted in allopatric speciation (Cadena et al. 2011). Preliminary results from a comparison between the amphibian communities from the Serranía de Majé and Serranía de Piedras-Pacora (Ibáñez et al. 1994, Sosa and Guerrel 2013), located across the valley of the Chepo River (Figure 1), showed a lower species diversity at the Serranía de Majé and a decrease in the similarity of species composition as elevation increases (Figure 4). In addition, the highest elevations studied at these two mountain ranges are about 106 km apart (airline distance), and their dissimilarity is largely due to the disproportionate number of species that are present in Serranía de Piedras-Pacora but potentially absent in Serranía de Majé. Hence, thus far, seems that dispersal limitation has potentially played a major role in shaping the amphibian community at Serranía de Majé; nonetheless, more studies would be necessary to address this. Lastly, the decrease in similarity in species composition as elevation increases is consistent with the general pattern of amphibians endemism observed in Central America, that shows that a substantial portion of endemic species in the region are associated with upland regions (Whitfield et al. 2016).

Central America, while being a hotspot for amphibian diversity, is a region with a high proportion of threatened amphibian species. For instance, 41% of the regional pool of species that have been assessed by the IUCN (International Union for Conservation of Nature) are under one of the following categories of the Red List of Threatened Species: critically endangered, endangered or vulnerable (reviewed in Whitfield et al. 2016). Within this context, the amphibian community of the Serranía de Majé does not seem, at first, to be comprised of species of high conservation concern given that 76% of the species registered in this study are under the category ‘least concern’ of the IUCN Red List of Threatened Species (IUCN 2018). However, the Serranía de Majé harbors amphibian species that could be regarded as threatened species, as well as poorly known species lacking an evaluation of their conservation status. For example, based on the IUCN criteria, two of the recorded species are considered near threatened (i.e., *Pristimantiscaryophyllaceus* and *P.pardalis*), and two others are data deficient (i.e., *Caeciliaisthmica*, and *Hyalinobatrachiumvireovittatum*). Importantly, these two species that are considered near threatened and the two data deficient ones are endemic to Central America (Johnson et al. 2015). Notably, in this study we also found four species, which include one species from the genus *Pristimantis* (P.aff.latidiscus), two dendrobatids (Colostethusaff.pratti and Silerstoneiaaff.nubicola) and one species from the *Diasporusdiastema* species group (i.e., Diasporusaff.diastema suggested by Batista et al. 2016), that are potential new species and, together with the recently described *Diasporusmajeensis*, lack an assessment by the IUCN.

Our survey provides baseline information for exploration and conservation efforts by identifying species in the area requiring immediate assessment and conservation action (Table 1). Importantly, this study might also inform the delimitation of protected areas based on species with restricted distribution ranges. This is particularly relevant given the absence of biological reserves within this mountain range that are recognized by the national system of protected areas (Jaramillo et al. 2010), and the increasing deforestation pressure in the region (Parker et al. 2004). Lastly, considering the arrival of *Bd* to the Serranía de Majé some years after this study (Küng et al. 2014), the baseline information provided by this inventory could potentially serve to determine *Bd*-induced changes in the amphibian community. In particular, at mid and high elevations, where disease-induced losses of amphibian diversity have been substantial in Central America, including Panamá (Lips 2016).

## Supplementary materials

Supplementary material 1 Table S1. List of voucher specimens collected at the three surveyed sites along the altitudinal gradient in the Serranía de Majé Data type: specimen list. Explanation note: The list includes taxonomy of voucher specimens and collection number, date and locality data. CH = Collection of Herpetology, AJC = Andrew J. Crawford field tag, and MVUP = Museo de Vertebrados de la Universidad de Panamá. This dataset is made available under the Open Database License (http://opendatacommons.org/licenses/odbl/1.0/). The Open Database License (ODbL) is a license agreement intended to allow users to freely share, modify, and use this Dataset while maintaining this same freedom for others, provided that the original source and author(s) are credited.Daniel Medina, Roberto Ibáñez, Karen R. Lips, Andrew J. Crawford
